# Pro-Apoptotic and Pro-Autophagic Properties of Cardenolides from Aerial Parts of *Pergularia* *tomentosa*

**DOI:** 10.3390/molecules27154874

**Published:** 2022-07-29

**Authors:** Stefania Martucciello, Gaetana Paolella, Antonio Massimiliano Romanelli, Silvia Sposito, Lucia Meola, Antonietta Cerulli, Milena Masullo, Sonia Piacente, Ivana Caputo

**Affiliations:** 1 Department of Chemistry and Biology, University of Salerno, 84084 Fisciano, Italy; gpaolella@unisa.it (G.P.); aromanelli@unisa.it (A.M.R.); ssposito@unisa.it (S.S.); icaputo@unisa.it (I.C.); 2 Department of Neuroscience, Catholic University of the Sacred Heart, 00168 Rome, Italy; lucia.meola1@unicatt.it; 3 Department of Pharmacy, University of Salerno, 84084 Fisciano, Italy; acerulli@unisa.it (A.C.); mmasullo@unisa.it (M.M.); piacente@unisa.it (S.P.); 4 European Laboratory for the Investigation of Food-Induced Diseases (ELFID), University of Salerno, 84084 Fisciano, Italy

**Keywords:** cardiac glycosides, Asclepiadaceae family, bioactive compounds, anticancer compounds, apoptosis, autophagy

## Abstract

*Pergularia tomentosa* L., a milkweed tropical plant belonging to the family Asclepiadaceae, is a rich source of unusual cardiac glycosides, characterised by transfused A/B rings and a sugar moiety linked by a double link, generating a dioxanoid structure. In the present report, five cardenolides isolated from the aerial parts of the plant (calactin, calotropin, 12β-hydroxycalactin, 12β,6′-dihydroxycalotropin, and 16α-hydroxycalotropin) were investigated for their biological effects on a human hepatocarcinoma cell line. Cell viability was monitored by an MTT assay. The occurrence of apoptosis was evaluated by detecting caspase-3 activation and chromatin fragmentation. The ability of these compounds to induce autophagy was analysed by monitoring two markers of the autophagic process, LC3 and p62. Our results indicated that all cardenolides had cytotoxic effects, with IC_50_ ranging from 0.127 to 6.285 μM. All compounds were able to induce apoptosis and autophagy, calactin being the most active one. Some of them also caused a reduction in cell migration and a partial block of the cell cycle into the S-phase. The present study suggests that selected cardenolides from aerial parts of *P. tomentosa*, particularly calactin, possess potentially desirable properties for further investigation as anticancer agents.

## 1. Introduction

Several natural compounds from plants receive great attention for their potential application to prevent and contrast cancer development or to support conventional therapies thanks to their high efficacy and often low toxicity to normal cells [1,2]. Interacting with several cellular targets, natural compounds may activate signalling pathways, finally leading to the apoptosis of cancer cells, sometimes engaging in autophagic or endoplasmic reticulum (ER)-stress responses [2,3,4,5].

Among emerging bioactive compounds explored for their anticancer properties, there are cardiac glycosides, a group of molecules displaying the ability to bind the main ion transport protein pump, the Na^+^/K^+^-ATPase [6]. They have long been and continue to be employed as positive ionotropic agents in congestive heart failure [7], but, more recently, the potential anticancer activities of some of them, for example, digitoxin, digoxin or ouabain, have been demonstrated in vitro and in vivo [8,9]. From the chemical point of view, cardiac glycosides present a steroid nucleus with a lactone moiety at position 17 and a sugar moiety at position 3. The type of lactone moiety allows defining two subgroups, cardenolides and bufadienolides, whereas further subfamilies are defined on the bases of the sugar moiety [8].

The Asclepiadaceae family represents a rich source of cardenolides with peculiar features [10]. The A/B rings of the steroid nucleus are transfused, giving more flat structures and a more potent binding ability to the Na^+^/K^+^-ATPase pump [11]. Moreover, the sugar moiety is linked at position 3 by a double link, thus generating a dioxanoid structure [8].

Several cardenolides have been isolated and characterised from roots and leaves of *Pergularia tomentosa* [11,12,13,14], a tropical herb species of the Asclepiadaceae family, native to the Middle East, Egypt and South Africa. Studies on cytotoxic, antiproliferative, and pro-apoptotic properties of cardenolides from *P. tomentosa*, as well as on their strong inhibitory activity of the Na^+^/K^+^-ATPase pump have been reported, highlighting their prospective usefulness in cancer management [11,13,15,16,17].

**Figure 1:**
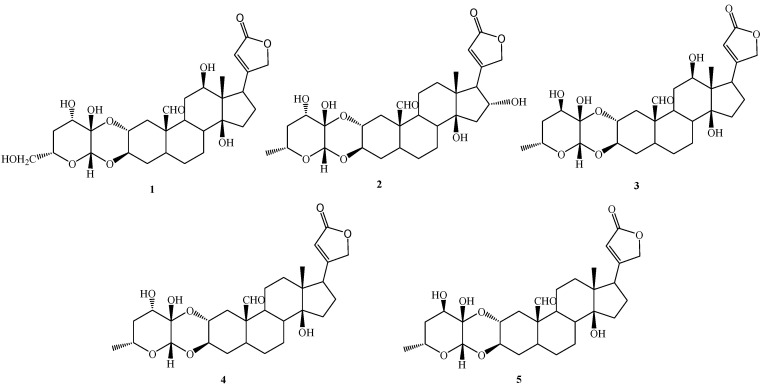
Cardenolides (**1**–**5**) isolated from the aerial parts of *Pergularia tomentosa*.

The present work aimed to investigate, in a deeper way, the potential anticancer properties of some cardenolides isolated from the aerial parts of *P. tomentosa* [13]. In particular, we focused attention on five selected compounds, three of which were identified in the roots and the aerial parts of *P. tomentosa*, i.e., 16α-hydroxycalotropin (**2**), calotropin (**4**), and calactin (**5**), while 12β,6′-dihydroxycalotropin (**1**), and 12β-hydroxycalactin (**3**) were only found in the aerial parts of *P. tomentosa* (Figure 1). In particular, compounds **1–3** were chosen for their high antiproliferative activity in PC3 cells [13]. Here, we investigated the cytotoxicity of these selected compounds in cancer cell lines not previously employed and explored their antiproliferative ability in several cell cultures and their pro-apoptotic and pro-autophagic activities in a hepatic cancer cell line.

## 2. Results

### 2.1. P. tomentosa Compounds Reduce Cell Viability in Cancer Cells

**Figure 2:**
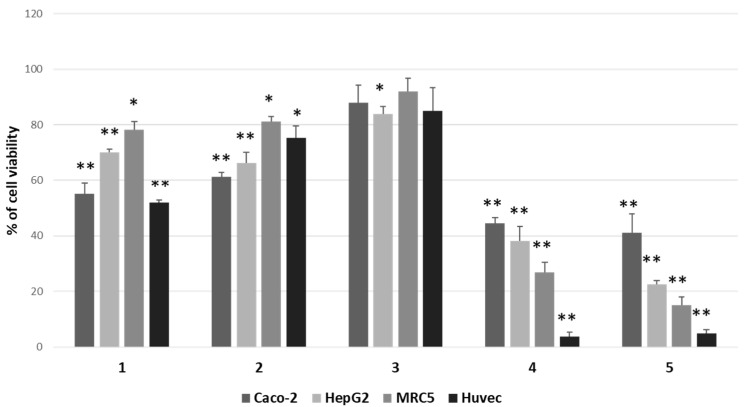
Comparison of cytotoxic effects of *P. tomentosa* compounds on cancer cell lines (Caco-2 and HepG2), on an immortalised cell line (MRC5) and normal cells (HUVEC). Cells were treated for 24 h with 1 μM of each compound. Residual viability was measured by the MTT assay. Values are the means ± standard error (SE) of three independent experiments performed in triplicate. Statistical analysis was performed using the Student’s *t*-test. * *p* < 0.05 and ** *p* < 0.01 vs. cells treated with the vehicle (DMSO). In each experiment, DMSO reduced cell viability by not more than 8%.

We focused attention on 12β,6′-dihydroxycalotropin (**1**), 16α-hydroxycalotropin (**2**), 12β-hydroxycalactin (**3**), calotropin (**4**), and calactin (**5**), among those described by Hosseini et al. [13], and tested their cytotoxic activity by a 3-(4,5-dime-thylthiazol-2-yl)-2,5-diphenylte-tetrazolium bromide (MTT) assay on two cancer cell lines (HepG2 and Caco-2), not previously employed, on an immortalised nontumoral cell line (MRC5) and normal primary cells (HUVEC). After treatments for 24 h with 1 μM of each compound, we found that the less cytotoxic one was compound **3**, whereas the most cytotoxic ones were compounds **4** and **5**, while compounds **1** and **2** displayed intermediate cytotoxicity (Figure 2).

The calculation of IC_50_ in HepG2, Caco-2 and MRC5 cells revealed that nontumoral cells were more sensitive than cancer cells towards compounds **1** and **2**, whereas they appeared similarly or less affected by other compounds (Table 1).

Moreover, by confronting Caco-2 cells and HepG2 cells, it was evident that these two cancer cell types were similarly affected by *P. tomentosa* compounds; however, HepG2 cells were more sensitive than Caco-2 cells to the cytotoxic activity of compound **5**. Consequently, we decided to perform the next analyses on the HepG2 cell line.

### 2.2. Effects of P. tomentosa Compounds on Cell Cycle Progression of HepG2 Cells

**Figure 3:**
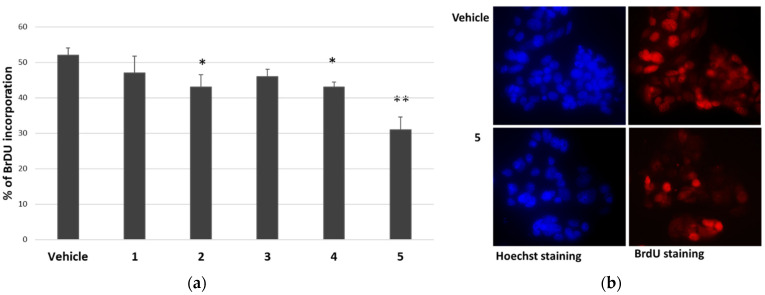
Effect of *P. tomentosa* compounds on BrdU incorporation in HepG2 cells. (**a**) Quantification of BrdU incorporation by cells cultured for 24 h in the presence of 1 μM of each compound. Data are reported as mean ± SE of three experiments. Statistical analysis was performed using the Student’s *t*-test. * *p* < 0.05, ** *p* < 0.01 vs. vehicle-treated cells. (**b**) Microscopic visualisation of representative Hoechst-stained (blue) and BrdU-stained (red) nuclei of cells treated with the vehicle or compound **5** (magnification 40×, with oil).

Considering the reduction in cell viability of cancer cells in the presence of *P. tomentosa* compounds, we investigated their effect on the cell cycle progression of HepG2 cells. Cells were treated for 24 h with 1 μM of each compound; then, the entry into the S phase was evaluated by performing a 5-bromo-2′-deoxy-uridine (BrdU) incorporation assay. In this assay, compounds **1** and **3** slightly (but not significantly) reduced BrdU incorporation, whereas compounds **2**, **4** and **5** significantly affected cell cycle progression (Figure 3).

**Figure 4:**
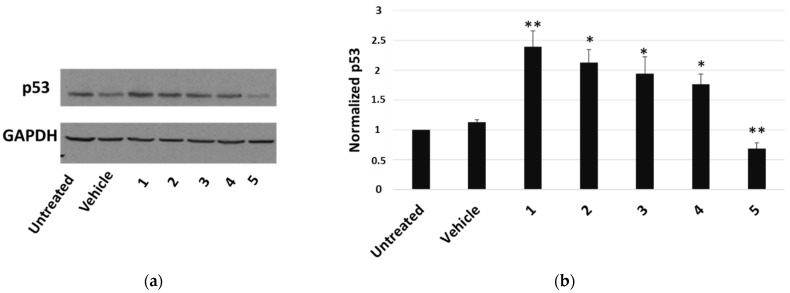
Western blot analysis of p53 expression in HepG2 cells. (**a**) Representative Western blot on 50 μg of total proteins from cells treated for 24 h with 1 μM of each compound or vehicle only. (**b**) Densitometric analysis relative to three independent Western blots. Protein levels are normalised with respect to GAPDH expression and reported as variation with respect to the untreated sample. Data are reported as mean ± SE of three experiments. Statistical analysis was performed using the Student’s *t*-test. * *p* < 0.05, ** *p* < 0.01 vs. vehicle-treated cells.

The most cytotoxic compound, calotropin (**4**), reduced by about 40% the number of cells that incorporated BrdU compared to the vehicle. We next investigated whether *P. tomentosa* compounds modulated the expression of p53, given the important role that this protein plays in controlling proliferation. After treatments for 24 h at 1 μM, we observed an increase in p53 protein level for all compounds, except compound **5**, which clearly decreased the p53 amount (Figure 4), indicating a quite different mechanism of this compound in regulating the cell cycle.

### 2.3. Influence of P. tomentosa Compounds on Cell Migration of HepG2 Cells

**Figure 5:**
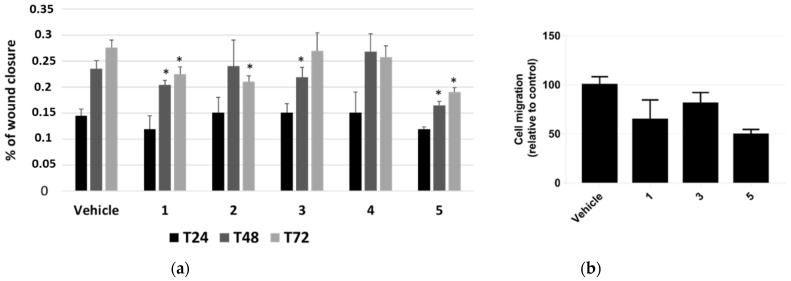
Scratch-wound-healing assay on HepG2 cells. (**a**) Analysis of the mean wound closure after treatments for 24, 48, and 72 h with *P. tomentosa* compounds at 1 μM. Data are reported as mean ± SE of three independent experiments, each in duplicate. * *p* < 0.05 vs. vehicle-treated cells. (**b**) Transwell migration assay in cultured HepG2 cells. Cell migration is reduced after transfection after treatments for 18 h with *P. tomentosa* compounds at 1 μM. Data are reported as mean ± SE of three independent experiments, each in duplicate. Statistical analysis was performed using the Student’s *t*-test.

The effect of *P. tomentosa* compounds on influencing cell migration was first investigated by performing a scratch-wound-healing assay. We took photos at 24, 48 and 72 h from the beginning of treatments with 1 μM of each compound and analysed data as described in the methods section. Even if the migration was not very pronounced, we could highlight slight but significant differences. Compounds **1** and **5** interfered with the wound closure after 48 and 72 h of treatment; compound **2** interfered with the wound closure at 72 h only, whereas compound **3** had an effect at 48 h only; compound **4** seemed to not affect cell migration, at any time (Figure 5a).

Based on these results, we further investigated HepG2 cells migration by performing a migration assay in transwell chambers for 18 h with 1 μM of compounds **1**, **3** and **5**. All compounds were able to reduce migration, with compound **5** being the most potent (Figure 5b). On the whole, data confirmed those from scratch-wound-healing assay.

### 2.4. Modulation of Apoptosis by P. tomentosa Compounds

**Figure 6:**
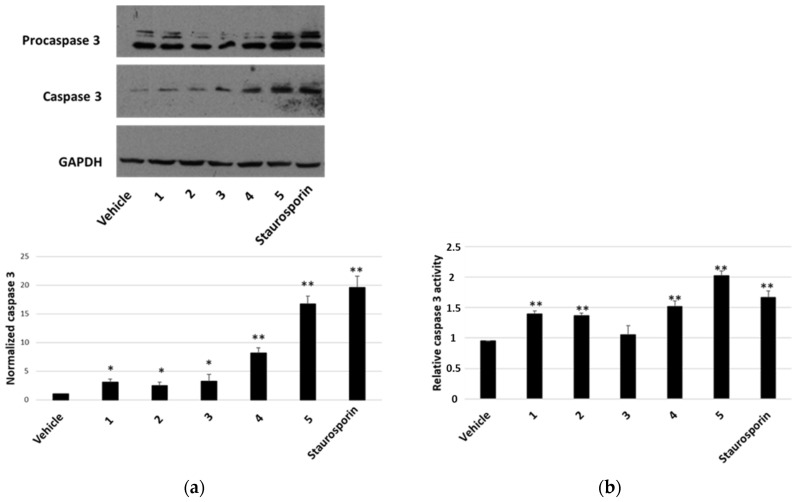
Caspase-3 activation in HepG2 cells. ((**a**), upper panel) Representative Western blot anti-caspase 3 on 70 μg of proteins from cells treated for 7 h with 2 μM of each compound or vehicle or staurosporin. ((**a**), lower panel) Densitometric analysis relative to cleaved caspase-3, performed on three independent Western blots. Protein levels are normalised with respect to the GAPDH expression and reported as variation with respect to the vehicle. Data are reported as mean ± SE. * *p* < 0.05 and ** *p* < 0.01 vs. vehicle-treated cells. (**b**) Relative caspase-3 activity, normalised towards activity measured in untreated cells. Data are reported as mean ± SE of three independent experiments. ** *p* < 0.01 vs. vehicle-treated cells.

**Figure 7:**
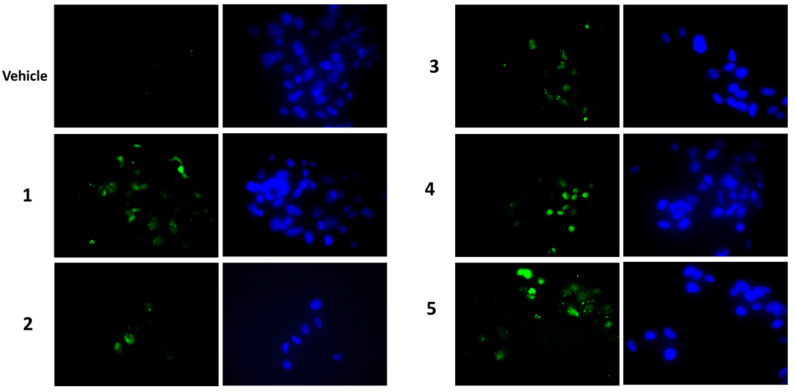
Microscopic detection of apoptosis by the TUNEL assay in HepG2 cells treated with 1 μM of each compound, or vehicle only, for 24 h. Apoptotic nuclei are in green (on the left), total Hoechst-stained nuclei are in blue (on the right). Magnification 40×, with oil.

To establish whether the cytotoxicity observed in the MTT assay was also related to increased apoptosis, the presence of activated caspase-3 by Western blot was analysed as well as the caspase-3 activity assay. After a treatment of 7 h with compounds at a concentration of 2 μM, we visualised the appearance of cleaved caspase-3 at the expected molecular weight for all compounds tested (Figure 6a). Densitometric analyses highlighted that all compounds were able to activate caspase-3 and compounds **4** and **5** were the most active ones (Figure 6a, lower panel). These findings were confirmed by the detection of caspase-3 activity, measured after a treatment of 24 h with 1 μM of each compound. In this case, an increase in caspase-3 activity was observed for all compounds (except for compound **3**) (Figure 6b). In all experiments, staurosporin was used as a positive control. When we performed a terminal deoxynucleotidyl transferase dUTP nick end labeling (TUNEL) assay (Figure 7), we clearly appreciated the occurrence of apoptotic nuclei in cells treated with compounds **4** and **5**; apoptosis was less evident in cells treated with compounds **1** and **2** and was undetectable in cells treated with compounds **3**.

### 2.5. Effects of P. tomentosa Compounds on ER-Stress Markers

**Figure 8:**
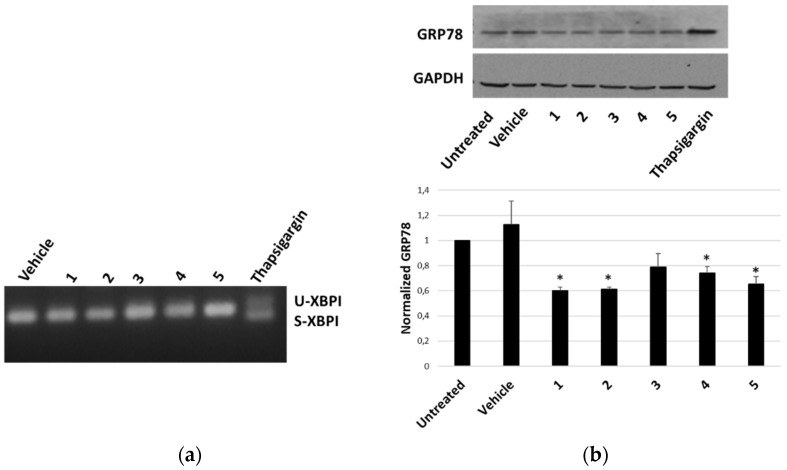
ER-stress markers in HepG2 cells treated with *P. tomentosa* compounds. (**a**) Agarose gel showing bands relative to unspliced (U) and spliced (S) forms of XBP1. ((**b**), upper panel) Representative Western blot of anti-GRP78 on 70 μg of proteins of cells treated with *P. tomentosa* compound for 24 h at 1 μM. ((**b**), lower panel). Densitometric analysis relative to three independent Western blots. Protein levels are normalised with respect to the GAPDH expression and reported as variation with respect to the untreated sample. Data are reported as mean ± SE. Statistical analysis was performed using the Student’s *t*-test. * *p* < 0.05 vs. vehicle-treated cells.

The ability of *P. tomentosa* compounds to trigger an ER response in HepG2 cells was first evaluated by analysing the occurrence of the splicing of X-box binding protein 1 (XBP1), an early marker of the unfolded protein response typical of a condition of ER stress. We observed the appearance of the spliced form only in the case of the treatment with thapsigargin, the positive control; instead, only non-spliced bands were evident after treatment with *P. tomentosa* compounds (Figure 8a). To confirm that an unfolded protein response was not engaged, we also analysed the protein level of another early marker of ER stress, i.e., the glucose-regulated protein-78 (GRP78). The results showed how the GRP78 expression did not increase after 24 h of treatment at 1 μM; on the contrary, a slight decrease in GRP78 levels for all compounds tested was evident (Figure 8b).

### 2.6. Modulation of Autophagy by P. tomentosa Compounds in HepG2 Cells

**Figure 9:**
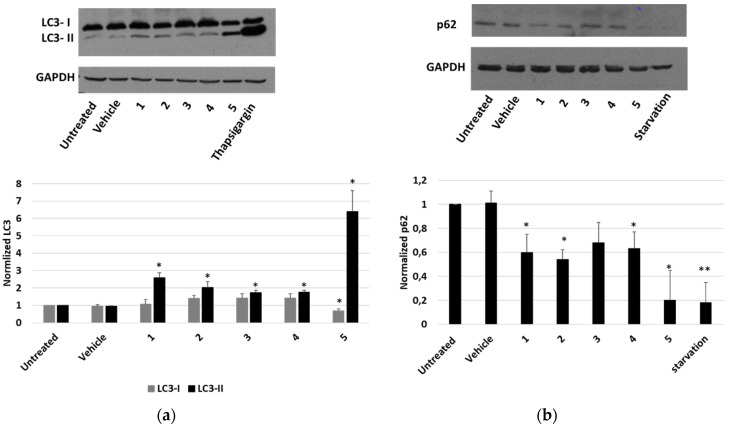
Expression of autophagic markers in HepG2 cells treated with *P. tomentosa* compounds. ((**a**), upper panel) Representative Western blot of anti-LC3 on 50 μg of proteins of cells treated with *P. tomentosa* compound for 24 h at 1 μM. ((**a**), lower panel) Densitometric analysis relative to LC3-I and LC3 II forms performed on three independent Western blots. ((**b**), upper panel) Representative Western blot of anti-p62 on 70 μg of proteins of cells treated with *P. tomentosa* compound for 4 h at 2 μM. ((**b**), lower panel) Densitometric analysis relative to p62 performed on three independent Western blots. In both a and b, protein levels are normalised with respect to the GAPDH expression and reported as variation with respect to the untreated samples. Data are reported as mean ± SE. Statistical analysis was performed using the Student’s *t*-test. * *p* < 0.05, ** *p* < 0.01 vs. vehicle-treated cells.

Finally, the occurrence of an autophagic pathway in cells treated with *P. tomentosa* compounds was investigated. To this aim, the modification of the level of the two forms of LC3 (LC3-I and LC3-II) after treatments was observed. As shown in Figure 9a, all compounds induced an increase in LC3-II. In the case of compound **5,** such an increase was more marked, and a reduction in the LC3-I form was also evident. In these experiments, thapsigargin was used as a positive control of the autophagy induction. To confirm that autophagy was occurring, we also evaluated the expression level of p62. We performed a Western blot analysis after 4 h of treatment with 2 μM of each compound. Starvation was used as a positive control to detect the reduction in p62 level (Figure 9b). The results showed that all compounds reduced p62 level, indicating the occurrence of a normal autophagic flux.

## 3. Discussion

In the present work, we investigated the mechanism at the bases of cytotoxicity of some cardenolides obtained from leaves of *P. tomentosa*. Cardenolides, a subgroup of cardiac glycosides, are abundantly present in the roots and aerial parts of several plants [14]. The anticancer properties of cardenolides (and, more in general, of cardiac glycosides) have been studied in different cell models, and there is a lot of evidence of their efficacy [8,18,19]. The mechanisms of action of these phytochemicals may include the inhibition of proliferation, the induction of apoptosis or autophagy and the sensitisation to chemotherapy [9,10,20]. Previous studies performed with cardenolide-rich extracts or pure cardenolides from *P. tomentosa* demonstrated that they not only exerted a cardiotonic activity [21] but also reduced the cell viability of cancer cells, had a pro-apoptotic effect on Kaposi’s sarcoma cells and exerted an antiangiogenic effect both in in vitro and in vivo models [11,12,13,17,22]. The inhibition of Na^+^/K^+^-ATPase activity has been considered the possible main mechanism responsible for the anticancer properties of these bioactive compounds [12].

In our work, we focused on five doubly linked cardenolides, a subclass of cardenolides found in *Pergularia* genus [23]. These compounds were selected from a bigger group already tested in a previous work by Hosseini et al. [13] demonstrating that known cardenolides, together with new isolated ones, were able to decrease the cell viability of five different human cancer cell lines. Interestingly, IC_50_ for each compound was very variable from one cell line to another, indicating that different cancer cells presented their sensitivity towards these compounds [13].

Generally, in cardiac glycosides isolated from plants belonging to the genus *Digitalis* (family Scrophulariaceae) and *Strophanthus* (Apocynaceae), rings A/B and C/D are *cis* fused, while rings B/C are *trans* fused [24,25]. Such ring fusion gives the aglycon nucleus of these cardiac glycosides a characteristic “U” shape [11]. On the other hand, in cardiac glycosides produced by plants from the family Asclepiadaceae, such as *Pergularia*, the A/B rings are *trans* fused, resulting, thus, in rather flat structures. It is worth noting that, whereas cardiac glycosides from *Digitalis* and *Strophanthus* species contain sugar units linked through the 3β-OH of the steroid aglycon (single link), compounds produced by plants from the Asclepiadaceae are characterised by a sugar moiety linked to the 2α- and 3β-positions of the aglycon by hemiacetal and acetal functions, respectively, generating a “dioxanoid” structure [11,13]. The sugar unit can be represented by a 4,6-dideoxyhexosulose (**1**) and a modified form of the latter, 4-deoxyhexosulose (**2–5**). Moreover, compounds **1–5** can be considered as belonging to two groups: the calotropin derivatives (**1**, **2** and **4**) and the calactin derivatives (**3** and **5**), differing in the configuration at C-3′. These structural features are rarely found in cardenolides from families different from Asclepiadaceae.

An MTT assay analysed the cytotoxic activity of these compounds in two cancer cell lines not previously tested, i.e., HepG2 and Caco-2. Moreover, an immortalised nontumoral cell line (MRC5) and a culture of normal cells (HUVEC) were used. Our findings showed that compounds were cytotoxic towards all cells tested, compound **3** being the less active. Normal/noncancer cells and cancer cells were similarly affected by compounds **1**, **2** and **3**, whereas compounds **4** and **5** were more cytotoxic for normal/noncancer cells than for cancer cells. These findings indicate a non-specific action of the tested compounds toward cancer cells. However, intestinal- and hepatic-cancer-cell lines appeared very sensitive, particularly to the activity of calactin and calotropin. Calculated IC_50_ confirmed that each cell type displayed a different sensitivity and also revealed that HepG2 cells were more sensitive to the effect of calactin (**5**) but less sensitive toward the calactin derivative (**3**) than Caco-2 cells. Focusing the attention on HepG2 cells, all molecules used at 1 μM reduced the entry into the S-phase. The effect was more marked for compound **5**, in line with the result of MTT assays. The ability to reduce cell migration also highlighted the potential to interfere with signalling pathways in cancer cells.

Furthermore, also apoptosis could contribute to reducing cell viability. We detected an increase in the appearance of cleaved caspase 3 as a consequence of the treatment with each compound, with a more marked signal after treatments with compounds **4** and **5**. The caspase 3 activity assay confirmed this trend, even if compound **3**, at the condition tested, did not seem to affect enzyme activity. Microscopic visualisation of the presence of apoptotic nuclei by the TUNEL technique was in line with biochemical assays. The Western blot analysis on HepG2 samples showed an increase in the pro-apoptotic protein p53 for all compounds, except for calactin (**5**), which reduced the p53 level. This finding highlighted that calactin had a mechanism of action that, in part, was different from the other tested compounds.

Several natural products, including cardenolides, can induce an ER-stress response, that can cause a mechanism of programmed cell death [2,5,26]. However, on the contrary, natural products may also inhibit proteins related to the unfolded protein response, thus reducing the adaptative response of the cell [5]. Herein, two early markers of ER stress, XBP1 and GRP78, were investigated, but the activation of an unfolded protein response was not observed. Conversely, we observed a slight decrease in GRP78 level, which is compatible with this protein’s pro-survival role; indeed, in several conditions, a reduction in GRP78 has been associated with increased apoptosis [27,28].

A desirable feature of an anticancer drug may be the ability to modulate autophagy [1,4]. Autophagy is a process that, at the basal level, promotes cellular homeostasis, but in stressful conditions, it may act as a protective response or a pro-death pathway, depending on the cell type, the environmental context and the nature of the stressor [29]. The pro-death role of autophagy can occur by inducing the apoptotic mechanism or without the involvement of the apoptotic machinery [2,29,30]. Several cardiac glycosides have displayed a pro-autophagic activity as part of their anticancer effect [31,32,33]. We found that *P. tomentosa* compounds induced autophagy, as highlighted by the reduction in p62 level and the appearance of the LC3-II form. The phenomenon was more evident for calactin than for other compounds. This could be the reason of the reduction in p53 level by calactin, in line with the reported suppression of p53 by autophagy [30,34]. A reduction in p53 protein synthesis has been already reported as a mechanism to mediate the anticancer effects of other cardiac glycosides [35]. Thus, we attest to the induction of apoptosis and autophagy in the same cell culture, and autophagy could precede apoptosis [30].

## 4. Conclusions

On the whole, our findings highlight an evident cytotoxic activity of cardenolides from the leaves of *P. tomentosa* towards cancer cells, confirming and expanding previously reported data. Calactin (**5**) is the most active compound in all assays and cells tested, whereas its derivative (**4**) is the less active, even if the two compounds have only minimal structural differences. The cytotoxicity mechanism in HepG2 cells involves both autophagy and apoptosis. These results suggest that the cardenolides from aerial parts of *P. tomentosa*, particularly calactin (**5**), possess potential desirable properties to be further investigated as anticancer agents, at least for some tumours.

## 5. Materials and Methods

### 5.1. Extraction and Isolation Procedures of Compounds **1**–**5** from the Aerial Parts of P. tomentosa

Aerial parts of *P. tomentosa* L. were collected near Nehbandan, Iran, in April 2018. The aerial parts of *P. tomentosa* (70 g) were dried and extracted at room temperature using solvents of increasing polarity including hexane (0.5 L for 3 days, two times), CHCl_3_ (0.5 L for 3 days, two times), and MeOH (0.5 L for 3 days, three times). After filtration and evaporation of the solvent to dryness in vacuo, 3 g of a crude MeOH extract was obtained. The extract was fractionated using a Sephadex LH-20 (Pharmacia) column (100 × 5 cm), with MeOH as mobile phase, affording 53 fractions (8 mL), as monitored by TLC. Fractions were analysed by an RP-HPLC-UV system. The elution gradient was obtained using water with 0.1% formic acid as eluent A and acetonitrile with 0.1% formic acid as B at a flow rate of 2.0 mL/min. For fractions 13−15 (950 mg), the HPLC gradient started at 10% B, and after 10 min % B was at 30%; after 16 min, it was at 54%, after 7 min, it was at 63%; and after 11 min, it was at 100%, holding it for 10 min. In this way, the compounds were as follows: **1** (3.4 mg, t_R_ = 14.25 min), **2** (4.2 mg, t_R_ = 16.20 min), **3** (4.5 mg, t_R_ = 18.36 min), **4** (4.5 mg, t_R_ = 22.90 min), and **5** (3.0 mg, t_R_ = 24.82 min). Compounds **1−5** were identified by comparison of their ^1^H and ^13^C NMR data with those reported previously in the literature [12]. NMR spectroscopic data were acquired in MeOH-*d_4_* (99.95%, Sigma-Aldrich, St. Louis, MO, USA) on a Bruker DRX-600 spectrometer (Bruker BioSpin GmBH, Rheinstetten, Germany) equipped with a Bruker 5 mm TCI CryoProbe at 300 K. Data processing was carried out with Topspin 3.2 software. The purity of these compounds (>99%) was determined by HPLC analysis. Stock solutions of *P. tomentosa* compounds were prepared in dimethyl sulfoxide (DMSO) and stored at −20° C in small aliquots.

### 5.2. Cell Cultures

The human liver hepatocellular carcinoma cell line, HepG2, and the human adenocarcinoma of colon cell line, Caco-2, were cultured in Eagle’s Minimum Essential medium supplemented with 1% (*v*/*v*) non-essential amino acids, 0.2 mM L-glutamine, 50 units/mL penicillin, 50 μg/mL streptomycin and 10% (*v*/*v*) or 20% (*v*/*v*) fetal bovine serum (Sigma Aldrich, Milan, Italy) for HepG2 or Caco-2, respectively. The human embryonal lung fibroblast cell line, MRC5, was cultured in Dulbecco’s Modified Eagle medium (Sigma Aldrich, Milan, Italy) supplemented with 10% (*v*/*v*) fetal bovine serum, 0.2 mM L-glutamine, 50 units/mL penicillin and 50 μg/mL streptomycin. HUVEC cells were cultured in endothelial growth medium-2, supplemented with fetal bovine serum (2%), VEGF (0.1%), rH FGF-B (0.4%), rH EGF (0.1%), GA-1000 (0.1%), hydrocortisone (0.04%), R3-IGF-1 (0.1%), heparin (0.1%) and ascorbic acid (0.1%). Cells were maintained at 37 °C in a 5% CO_2_, 95% air-humidified atmosphere and passaged twice a week. Cell lines were obtained from Interlab Cell Line Collection, Istituto Nazionale per la Ricerca sul Cancro, Genoa, Italy. HUVEC were obtained from Lonza (Milan, Italy).

### 5.3. Cell Viability Assay and IC_50_ Calculation

An MTT assay was used to analyse cell viability. Cells were seeded in 96 wells at the density of 2 × 10^4^/cm^2^ and, after 24 h, they were treated with *P. tomentosa* compounds or with the vehicle (DMSO). After a further 24 h of treatment, 0.5 mg/mL of MTT was added to the cell medium and incubated for 1.5 h at 37 °C. The resulting formazan crystals were dissolved in 100 μL of DMSO and absorbances were measured at 595 nm and 655 nm. The background signals at 655 nm were subtracted from 595 nm signals, and data were expressed as a percent of cell viability. The concentration of each compound that is required for 50% inhibition in vitro (IC_50_) was calculated as described in Husseini et al. [12]. IC_50_ values were obtained for Caco-2, HepG2 and MRC5 cells by performing MTT assays with concentrations ranging from 0.01 μg/mL to 5 μg/mL.

### 5.4. Proliferation Assay

Cell proliferation was analysed by a BrdU (Roche Diagnostics SpA, Monza, Italy) incorporation assay, as previously described [25]. Briefly, HepG2 cells, seeded at adensity of 3 × 10^3^/cm^2^ on round glass cover slips, were treated for 24 h with *P. tomentosa* compounds at 1 μM. Then, BrdU was added to cell medium (final concentration, 100 μM) for 1 h. Cells were fixed (in 3% paraformaldehyde, 10 min), permeabilised (in 0.2% Triton X-100, 5 min) and treated with HCl 1.5 N (5 min). Finally, cover slips were incubated with a monoclonal antibody against BrdU (Sigma-Aldrich, Milan, Italy), 1:100, and with a TRITC-conjugated secondary antibody, 1:100. After staining with Hoechst, cover slips were mounted with Mowiol (Sigma, Milan, Italy). Stained cells were observed with an AxioSkop 40 fluorescent microscope (Carl Zeiss MicroImaging, Inc., Jena, Germany). Images were acquired with Axiocam MRc5 and processed with ImageJ Launcher. The number of cells into S-phase was expressed as the ratio between the number of cells incorporating BrdU and the total number of cells.

### 5.5. Scratch-Wound-Healing Assay

Cells were seeded at a density of 5 × 10^4^ cells/well in 24-well plates and allowed to grow for 72 h, reaching confluence. Mitomycin C (10 μg/mL) was added to inhibit proliferation, and then a sterile 2–10 μL pipette tip was used to perform a vertical scratch in the monolayer of each well. Detached cells were removed by washing with phosphate-buffered saline. Then, 300 μL of fresh medium containing *P. tomentosa* compounds (1 μM) or vehicle was added to each well and incubated for 72 h. Images of migrating cells were taken at 0, 24, 48 and 72 h, by using an Olympus CKX41 fluorescent microscope (Olympus Italia srl, Segrate, Italy) and elaborated by the ImageJ software. For each scratch, wound width was calculated as the average distance between the edges in four different sites. Then, reductions in wound wights at 24, 48 and 72 h were expressed as % of the width at time zero.

### 5.6. Transwell Migration Assay

Transwell filters (coated with collagen) in serum-free media containing 0.1% of BSA, with a PET membrane with 8 μm pores (BD Biosciences, Milan, Italy), were rehydrated for 2 h at 37 °C. HepG2 were seeded in the upper chambers at a density of 75 × 10^3^ cells in MEM with 0.1% BSA and treated with or without 1 µM of *Pergularia* compounds. MEM−0.1%BSA, supplemented with 10% FBS, was added to the lower chamber as chemoattractant. Control wells without 10% FBS were included to assess random migration. After incubation at 37 ^°^C in 5% CO_2_ for 18 h, the migrated cells were fixed with 4% paraformaldehyde for 5 min, permeabilised with methanol for 20 min and stained with crystal violet 0.5% for 15 min at room temperature. The cells on the lower side of the filter were allowed to dry before counting. Eight separate bright-field images were randomly acquired of each filter using an Olympus CKX41 Image Analyzer. The cells in each image were counted and analysed compared to control-transfected cells.

### 5.7. Caspase-3 Assay

To evaluate apoptosis, a caspase-3 colorimetric assay was performed. Briefly, HepG2 cell lines were seeded in plates of 60 mm diameter at a density of 3.5 × 10^4^/cm^2^ and, after 24 h, treated with *P. tomentosa* compounds (1 μM each) for 24 h. Then, cells were harvested in phosphate-buffered saline, collected by centrifugation and lysed in a buffer containing 50 mM HEPES, 0.1% CHAPS, 10 mM dithiothreitol, 100 mM NaCl, 1 mM EDTA and 10% sucrose. Proteins (30 μg) were incubated for 2 h at 37 °C in a reaction mixture containing the caspase-3 substrate (acetyl-Asp-Glu-Val-Asp, labelled with p-nitroanilide) (Sigma-aldrich, Milan, Italy) at a concentration of 0.2 mM. Finally, the release of free p-nitroanilide was monitored spectrophotometrically at 405 nm. Enzyme activity was expressed as percentage of absorption of treated cells versus vehicle-treated cells.

### 5.8. Western Blot Analyses

To analyse the protein levels of p53, caspase-3, GRP78, LC3 and p62, HepG2 cells were treated with *P. tomentosa* compounds (1 or 2 μM) or with DMSO for the indicated times; then, Western blot analyses were performed. Briefly, cells were lysed in RIPA buffer (20 mM Tris-HCl, pH 7.5, 150 mM NaCl, 1 mM EDTA, 1 mM dithiothreitol, 0.1% sodium dodecyl sulphate, 1% triton X-100, 1 mM orthovanadate, and a cocktail of inhibitors (Sigma-Aldrich, Milan, Italy)). After separation on a sodium dodecyl sulphate-polyacrylamide gel electrophoresis and transfer to a poly-vinylidene fluoride membrane (Euroclone, Milan, Italy), the following primary antibodies were used, at a dilution of 1:1000 in tris-buffered saline containing 1% non-fat dry milk, overnight at 4 °C: mouse anti-p53 antibody, mouse anti-MAP LC3 β antibody, mouse anti-p62 antibody (DBA, Milan, Italy), mouse anti-GRP78 antibody, and mouse anti-caspase 3 antibody (Thermo Fisher Scientific, Milan, Italy). For normalisation, a mouse anti-GAPDH antibody (Microtech, Naples, Italy) was used. A horseradish-peroxidase-conjugated anti-mouse secondary antibody (Bio-Rad laboratories S.r.l, Milan, Italy) was used for 1 h; finally, immunocomplexes were revealed using a chemiluminescence detection kit (Microtech, Naples, Italy) according to the manufacturer’s instructions.

### 5.9. TUNEL Assay

Microscopic evaluation of apoptosis was performed by a TUNEL assay, using a commercial kit (Fragment End Labelling (FragEL™), Jena, Germany) DNA Fragmentation Detection Kit, Sigma-Aldrich, Milan, Italy) according to the manufacturer-provided protocol, with some modifications. Briefly, cells were seeded on glass cover slips placed in 24-well plates at a density of 3.5 × 10^3^/cm^2^ and, after 48 h, they were treated with 1 μΜ of each compound for a further 24 h. Cells were fixed in 3% paraformaldehyde for 10 min, permeabilised in 0.2% Triton X-100 for 5 min and treated with HCl 1.5 N for 8 min. Finally, cover slips were equilibrated in equilibration buffer (10 min) and incubated with label mix containing the TdT enzyme for 1 h at 37 °C, then mounted with mounting media. Microscope observations were performed by the AxioSkop 40 fluorescent microscope. Images were acquired with Axiocam MRc5 (Carl Zeiss MicroImaging, Inc., Jena, Germany).

### 5.10. XBP1 Splicing Detection

To detect the unspliced and the spliced form of XBP1, we followed the protocol described by Martucciello et al. [25]. After treatments for 4 h with 1 μM of each compound, or DMSO, or thapsigargin (1 μM), RNA was isolated and retro-transcripted. PCR analyses were performed with the following primers: 5′- CCTGGTTGCTGAAGAGGAGG-3′; 5′-CCATGGGGAGATGTTCTGGAG-3′. After 35 cycles (heating at 94 °C for 30 s, annealing at 58 °C for 45 s, and polymerisation at 72 °C for 60 s), amplified cDNA was visualised on 2.5% agarose gel, stained with an ethidium bromide solution.

### 5.11. Statistics

All data were expressed as means ± standard error (SE) of at least 3 independent experiments conducted in triplicates. Statistical analysis was performed using the Student’s *t*-test. In all experiments, differences were considered to be statistically significant at *p* < 0.01 or at *p* < 0.05.

## Figures and Tables

**Table 1:** Values of IC_50_ (μM) for Caco-2, HepG2 and MRC5 cells treated for 24 h with different concentrations of compounds. Each SE value (not shown) is less than 10% of the calculated IC_50_ value.

Compound	Caco-2	HepG2	MRC5
**1**	1.538	2.610	2.210
**2**	2.429	2.340	2.315
**3**	4.507	6.285	5.980
**4**	0.767	0.830	0.424
**5**	0.650	0.127	0.990

## Data Availability

Data not presented in this manuscript are available on request from the corresponding author.

## Sample Availability

Samples of compounds **1**–**5** are available in the authors’ lab.
